# A long forgotten ureteral stent for 13 years post renal transplantation

**DOI:** 10.1016/j.eucr.2022.102156

**Published:** 2022-07-04

**Authors:** Saad Mohammed Alwesali

**Affiliations:** Department of Urology, King Faisal University, College of Medicine, Al Ahsa, Saudi Arabia

**Keywords:** Stent, Kidney, Forgot, Encrustation

## Abstract

Stents have been promoted as an addition to the reconstruction of the urethra in kidney transplants. However, stents can cause serious complications, including migration, congestion obstructive uropathy. We present unusual case of long forgotten Double J-stent 13 years post renal transplant in absence of encrustation. A 43-year-old male patient was admitted with a history of colicky abdominal pain and diarrhea for three days. X-ray of the kidney and urinary bladder revealed retained Double J-stent stent. He was successfully treated by nephrostomy for the removal of the stent. Close follow-ups are necessary to avoid incidence of forgotten DJ stent.

## 1 Introduction

Due to advances in surgical procedures and experience, the prevalence of urological complications after kidney transplantation have dropped significantly during recent years. Placements of ureter stents can play a preventive role by reducing the urological complications following kidney transplant. Several advantages of stents include persistent decompression of the ureter to avoid anastomotic tension, maintenance of the ureter in a more linear alignment to avoid kinking, and protection from ureteral constriction or postoperative luminal obstruction due to edema or external compression. In fact, when double-J (DJ) stents are utilized with ureteroneocystostomy, the rate of serious urological complications dropped to nearly 2%–5%.^1^ However certain significant problems such as migration and encrustation leading to obstructive uropathy are associated with them and also stents may possibly be forgotten. We present an unusual case of long forgotten DJ stent of 13 years post renal transplant in the absence of encrustation. Comparison with the available literature data revealed, this is the longest documented time period for a forgotten ureteric stent in a patient of transplant without encrustation.

## 2 Case presentation

**Fig. 1:**
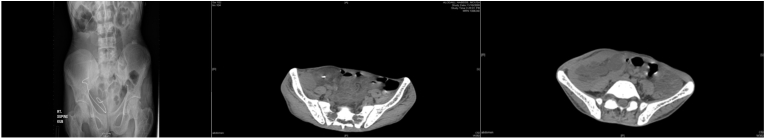
Moderate to severe hydronephrosis with displacement of DJ down to upper ureter with no evidence of encrustation.

**Fig. 2:**
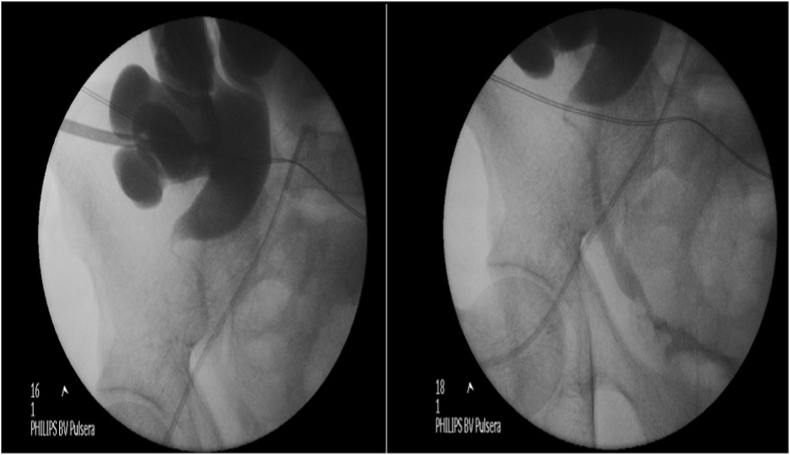
Antegrade pyelogram showed around 3 cm upper ureteric strictures.

**Fig. 3:**
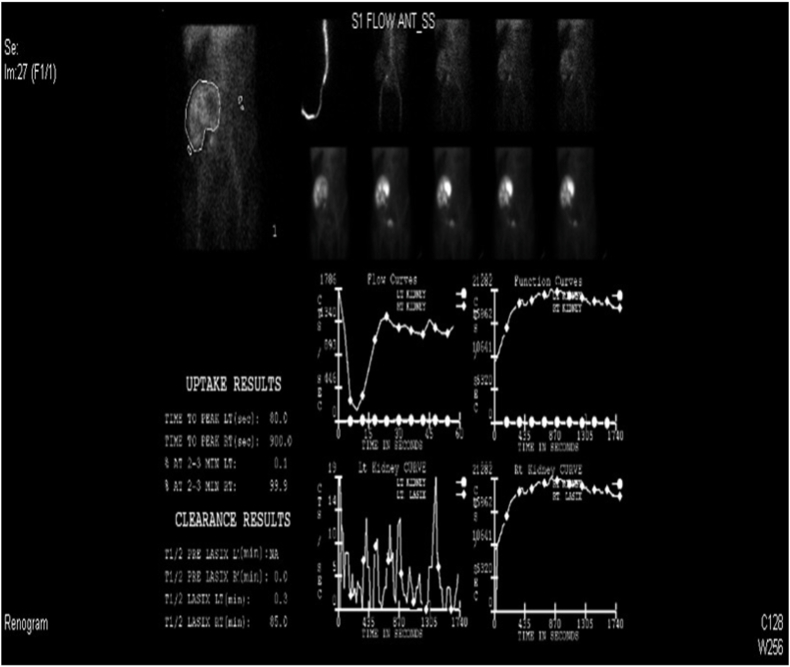
Diuretic renogram revealed incomplete obstruction.

A 43-year-old male patient who is a known case of sickle cell anemia underwent renal transplant 13 years ago in India for end staged renal disease of unknown etiology. The patient is on tacrolimus and azathioprine. He was on regular follow up in nephrology clinic five years ago when he was admitted to the intensive care unit with history of colicky abdominal pain and diarrhea that lasted for three days. He was diagnosed as enteritis with dehydration and acute renal insufficiency with provisional diagnosis of urosepsis. On examination, the patient was lethargic, dehydrated and pallor with non-tender palpable transplanted kidney. Urine analysis showed leukocytes and increased red cells**.** X-ray of the kidney and urinary bladder revealed retained DJ stent. Computed tomography Scan showed moderate to severe hydronephrosis with displacement of DJ down to upper ureter with no evidence of encrustation (Fig. 1). Nephrostomy was performed and antegrade pyelogram showed around 3 cm upper ureteric strictures (Fig. 2). Two weeks later, the patient became hemodynamically stable and underwent a DJ stent removal without any stenting due to stricture. His clinical condition improved, and his creatinine dropped to 298 μmol/. Diuretic renogram revealed incomplete obstruction (Fig. 3). Antegrade DJ catheter was inserted, and his condition improved further with creatinine reaching 172 μmol/and the patient was voiding freely. After 3 months, the DJ stent was removed due to partial obstruction. Finally, a permanent pyeloplasty was done and patient had no complications for a duration of 5 years.

## 3 Discussion

Since almost four decades the ureter stents are in use and with the advancement in technology their application has broadened. Relief of obstructed pyelonephritis, bilateral ureteral obstruction, obstruction of a single functioning kidney, ureteric injury, and posttreatment of urolithiasis in patients with solitary confinement kidney are all absolute indications for stent implantation.^2^ They are also used as in adjunct treatment in kidney transplant patients. However, serious problems are associated with their use, especially if they are forgotten. These include migration, disintegration, overcrowding, and stone formation, leading to obstructive nephropathy which is a potentially dangerous outcome in a single kidney function.^3^ One of the most serious and common complications of the forgotten stent is stent encrustation. Additionally, life-threatening complications such as renal failure and urosepsis can ensue from urinary blockage caused by stent encrustations. Encrustations that form in DJ stents after being used for a long time or forgotten can cause complications during stent removal.^4^

Although there is no precise definition of a forgotten stent, the literature suggests stent left for more than six months is categorized as forgotten stent as it is to be removed from six weeks to six months depending upon condition. When a stent is left in place for 6 weeks, 6–12 weeks, or more than 12 weeks, the rates of encrustation are 9.2%, 47.5%, and 76.3%, respectively. Shock wave lithotripsy, ureteroscopy, and percutaneous nephrolithotomy, alone or in combination, may be required for comprehensive treatment of forgotten ureteral stents, especially those that have been in place for more than a year. DJ stents are a double-edged sword; if left for an extended period of time or forgotten, they can cause considerable morbidity in the patient. Furthermore, DJ stent can have many other complications such as obstruction, insufficient drainage, stone formation and migration.^5^ In our case, the double J catheter was kept in place for 13 years, interestingly there was no encrustation, and it was removed easily without breaking down. The only complication discovered in this patient is migration of the catheter down to upper ureter, we do not know when this happen. Also, we do not have an explanation for the absence of the encrustation and the continuity of the tensile power keeping the polymer molecules of the DJ catheter as one piece for this long period of time. Patient with sickle cell tend to have imbalance calcium to magnesium ration. Pathophysiologically, deposition of calcium and magnesium occurs on long indwelling stents. Therefore, it is hypothesized that encrustation did not develop due to the nature of this imbalance.

## 4 Conclusion

Long standing DJ catheter is quite a rare incidence. The unexpected findings to have no encrustations throughout the catheter duration of 13 years is unique. Also, the catheter could be removed as one unit without fragmentation. Close follow ups of such cases are required.

## Declaration of competing interest

None.
